# Capecitabine in combination with mitomycin C in patients with gastrointestinal cancer: results of an extended multicentre phase-I trial

**DOI:** 10.1038/sj.bjc.6602025

**Published:** 2004-07-06

**Authors:** R-D Hofheinz, J T Hartmann, A Willer, K Oechsle, G Hartung, U Gnad, S Saussele, S Kreil, C Bokemeyer, R Hehlmann, A Hochhaus

**Affiliations:** 1 Onkologisches Zentrum, III. Medizinische Klinik, Fakultät für Klinische Medizin Mannheim der Universität Heidelberg, Theodor-Kutzer-Ufer, D-68167 Mannheim, Germany; 2 Medizinische Klinik II, Universitätsklinikum Tübingen, Germany; 3 Abteilung Hämatologie und Onkologie, Klinik für Innere Medizin, Universität Rostock, Germany

**Keywords:** capecitabine, mitomycin C, gastrointestinal cancer

## Abstract

The aim of this study was to determine the dose-limiting toxicity (DLT) and establish the recommended dose for mitomycin C added every 3 weeks to the standard combination dose of capecitabine. Cohorts of at least three patients with pretreated gastrointestinal carcinoma received capecitabine 1000 mg m^−2^ orally twice daily on days 1–14 plus i.v. bolus mitomycin C on day 1 at doses of 4, 6, 8 or 10 mg m^−2^ (corresponding to dose levels I–IV). Cycles were repeated every 3 weeks. Two treatment cycles were considered for the evaluation of DLTs. Of the 53 patients enrolled, the majority had colorectal (*n*=27) or gastric (*n*=14) cancers. Patients had received a median of two lines of prior chemotherapy (34% with ⩾3 lines and 87% with prior 5-FU-based therapy). At the recommended dose level (IV, *n*=30), grade 3 adverse events during cycles 1 and 2 were: anaemia (10%); leukopenia (3%); thrombocytopenia (3%); stomatitis/mucositis (3%); hand–foot syndrome (3%). Two patients experienced DLTs (mucositis, *n*=1; neutropenic fever, *n*=1), but there were no grade 4 events. The median dose intensity for capecitabine and mitomycin C was 100% during cycles 1 and 2 and only four patients required postponement of therapy. Of the 43 patients evaluable for efficacy, seven achieved partial and minor remissions (16%; 95% CI, 5–28%), and 12 patients (28%) had stable disease. The favourable safety profile and promising activity of the capecitabine/mitomycin C combination, even in heavily pretreated patients, warrant further evaluation in patients with advanced colorectal and gastric cancers.

Front-line chemotherapy for patients with advanced/metastatic cancer of the digestive tract is frequently based on 5-fluorouracil (5-FU)±folinic acid (FA). Capecitabine is an oral fluoropyrimidine carbamate designed to mimic the pharmacokinetics of infusional 5-FU and produce high concentrations of 5-FU preferentially in tumour tissue. Capecitabine is converted to its active metabolite 5-FU through a three-step enzymatic process, the final step of which is mediated by the enzyme thymidine phosphorylase (Schüller *et al*, 2000). As first-line treatment for metastatic colorectal cancer, capecitabine produces a higher response rate than bolus 5-FU/FA and is at least equivalent in terms of time to disease progression (TTP) and overall survival (Twelves (2002)). In addition, capecitabine causes less diarrhoea, stomatitis, nausea, alopecia and neutropenia (Cassidy *et al*, 2002). Capecitabine has also demonstrated promising activity in other gastrointestinal cancers, as either a single agent or in combination with oxaliplatin (Borner *et al*, 2002), irinotecan (Tewes *et al*, 2003) or gemcitabine (Scheithauer *et al*, 2003).

Mitomycin C is one of the most effective single agents for gastrointestinal cancer. In addition, there is evidence for *in vitro* synergy between mitomycin and bolus 5-FU (Sartorelli and Boothe, 1965), although the cellular mechanism of the clinically observed synergy between mitomycin and infusional 5-FU has not been further elucidated. Nevertheless, time-dependent interactions between both drugs have been reported in human cancer cell lines (Rusello *et al*, 1989). Combination treatment with mitomycin C and bolus 5-FU/FA is both active and well tolerated in patients with advanced gastric cancer (Hartung *et al*, 2000). The addition of mitomycin to infusional 5-FU is also effective and well tolerated as first- or second-line therapy in patients with gastrointestinal tumours (Hartmann *et al*, 2003. Hofheinz *et al*, 2004). Mitomycin (10 mg m^−2^) added every third week to weekly infusional high-dose 5-FU/FA yielded a response rate of 54%, and an overall survival of 10.2 months in patients with advanced gastric cancer (Hofheinz *et al*, 2002). In a randomised study comparing ECF (three-weekly epirubicin and cisplatin with protracted venous infusional (PVI) 5-FU 200 mg m^−2^ daily) to MCF (mitomycin 7 mg m^−2^ every 6 weeks and three-weekly cisplatin with PVI-5-FU 300 mg m^−2^ daily) in patients with advanced oesophagogastric cancer, both regimens resulted in equivalent response rates and survival (Ross *et al*, 2002), thus confirming the efficacy of combined infusional 5-FU and mitomycin C. In patients with either colorectal or pancreatic cancer, PVI-5-FU plus MMC resulted in a superior response rate (and an improved TTP in colorectal cancer) compared with PVI-5-FU alone (Ross *et al*, 1997. Maisey *et al*, 2002).

As in the colorectal cancer setting, capecitabine is now replacing infusional 5-FU in combination with other agents such as mitomycin C due to its similar pharmacokinetic properties and superior tolerability and convenience. Furthermore, the antitumour activity of capecitabine is likely to be enhanced by the upregulation of thymidine phosphorylase activity by mitomycin C (Yamshita *et al*, 2001). Among various cytostatics studied in combination with capecitabine in human cancer xenografts, mitomycin C and the taxanes have the highest potential of increasing the levels of intratumoral thymidine phosphorylase (Sawada *et al*, 1998). The clinical relevance of upregulating thymidine phosphorylase has already been demonstrated for capecitabine in combination with docetaxel (±epirubicin) in patients with advanced breast cancer (O’Shaughnessy *et al*, 2002; Venturini *et al*, 2002).

The present phase I study was designed to evaluate the recommended dose and dose-limiting toxicities (DLTs) of capecitabine in combination with mitomycin C in patients with previously treated advanced gastrointestinal cancers.

## PATIENTS AND METHODS

The study protocol was reviewed and approved by the local institutional review boards of the participating institutions and the study was performed according to the Declaration of Helsinki. All patients provided written informed consent prior to entry into the study.

### Eligibility criteria

Patients with histologically confirmed gastrointestinal cancer refractory to or relapsed after at least one chemotherapy regimen for metastatic disease were eligible for entry into the study. Previous anticancer therapy was not permitted within the 4-week period immediately prior to study commencement. Other eligibility criteria included an Eastern Cooperative Oncology Group (ECOG) performance status ⩽2, age ⩾18 years, a life expectancy of ⩾3 months, and adequate bone marrow function (leucocyte count >3000 *μ*l^−1^, platelet count >100 000 *μ*l^−1^). Patients were also required to have adequate renal (serum creatinine ⩽1.4 mg dl^−1^ or creatinine clearance >60 ml min^−1^) and hepatic function (bilirubin ⩽2 mg dl^−1^). Left ventricular ejection fraction (LVEF) had to be ⩾50%. Patients of childbearing potential were required to be using appropriate contraception.

### Staging procedures

Before study admission, all patients underwent a complete history, physical examination, ECG and chest X-rays. Cardiac ultrasonography was carried out to determine the LVEF. A full blood count with differential and serum chemistry was obtained within 14 days prior to the start of treatment. Weekly blood counts were obtained and serum chemistry repeated every 3rd week or whenever clinically indicated. Assessment of LVEF was repeated before the initiation of every other treatment cycle. Computed tomography (CT) scans of the tumour-bearing region were recommended and performed within 4 weeks prior to the start of study treatment. Indicator lesions were assessed every 6 weeks.

### Treatment schedule and dose escalation

Capecitabine was administered orally within 30 min after a meal (generally after breakfast and evening meal) at a dose of 1 000 mg m^−2^ twice daily on days 1–14, every 3 weeks. Mitomycin C was administered by i.v. bolus on day 1 of each cycle at escalating doses (4, 6, 8 or 10 mg m^−2^, corresponding to dose levels I–IV). Dexamethasone 8 mg was added intravenously to mitomycin C to prevent pulmonary toxicity. All patients received standard antiemetic prophylaxis to avoid any bias relating to gastrointestinal toxicities.

Dose-limiting toxicities during the first two cycles of chemotherapy were defined by the occurrence of one of the following toxicities: grade 4 leucopenia/neutropenia or thrombocytopenia, symptomatic thrombocytopenia (haemorrhage), grade 3 or 4 febrile neutropenia or any ⩾grade 3 nonhaematological toxicity except nausea/vomiting. At least three patients were enrolled per dose level, with this number being increased to six if DLTs occurred in more than one patient. Dose escalation was halted if DLT occurred in two or more patients. The maximum tolerated dose (MTD) was defined as the highest dose at which fewer than two or three of six patients experienced DLTs during the first course of chemotherapy. Escalation beyond dose level IV was not foreseen and individual dose escalation was not allowed.

To adequately determine the safety of this combination, the recruitment of further patients at the MTD or at the highest investigated dose level (IV) was planned in the study protocol.

### Safety and efficacy analyses

Adverse events were recorded weekly and graded according to National Cancer Institute Common Toxicity Criteria (NCI-CTC, version 2.0). In terms of efficacy, complete response (CR), partial response (PR), minor response (MR), stable disease (SD) and progressive disease (PD) were defined according to WHO criteria. Survival was defined as the time between the start of chemotherapy and death, according to the Kaplan–Meier method (Kaplan and Maier, 1958).

### Statistical considerations

The rate of DLTs during the expanded study phase was required to be lower than 17% (i.e. about one out of six of the patients). With a total of 32 patients, a rate of at least 83% of the patients experiencing no DLTs during cycles one and two can be excluded using Simon's two-stage design with a power of 80% and an *α*-value of 0.2 if less than five out of 15 (first stage) or eight out of 32 patients (second stage) experienced DLTs.

## RESULTS

### Patient characteristics

A total of 53 patients were enrolled at three institutions between November 2001 and January 2003. Of these, 49 were evaluable for safety. The remaining four patients were withdrawn for the following reasons: lost to follow-up (*n*=1); violation of inclusion criteria (*n*=1); therapy discontinuation after 1 week due to ileus in need of surgery (*n*=1); and port infection with septic fever and need of port revision (*n*=1).

Characteristics of enrolled patients are described in Table 1

**Table 1:** Patient demographics

**Variable**	**No. of patients (%)**
Enrolled	53 (100)
Evaluable	49 (92)
	
*Age (years)*	
Median	61
Range	38–76
	
*Gender*	
Male	35 (66)
Female	18 (34)
	
*Performance status (ECOG)*	
Median	1
0	8 (15)
1	32 (60)
2	13 (25)
	
*Primary tumour site*	
Colorectal cancer	27 (51)
Gastric cancer	14 (26)
Pancreatic cancer	5 (9)
Oesophageal cancer	4 (8)
Others	3 (6)
	
*Prior chemotherapy*	
0–1 line	18 (34)
2 lines	17 (32)
⩾3 lines	18 (34)
5-FU based (i.v. or oral)	46 (87)
	
*Tumour localisation*	
Primary	14 (26)
Liver	35 (66)
Lung	24 (45)
Lymph nodes	16 (30)
Peritoneal carcinomatosis	14 (26)

. The majority of patients (85%) had an ECOG status ⩾1. Colorectal cancer (51%) and gastric cancer (26%) were the most common malignancies. The liver and lung were the most common sites of metastases, followed by lymph nodes and peritoneal carcinomatosis. Patients with colorectal cancer had received a median of three lines of prior chemotherapy and 87% of patients had received prior 5-FU-based treatment.

### Determination of the MTD and dose intensity

Three patients were enrolled at dose level I, seven at dose level II, nine at dose level III and 34 at dose level IV. Patients treated at dose levels I–III received a total of 31 cycles of chemotherapy. As allowed – but not formally requested – by the protocol, the number of patients treated at dose levels II and III was expanded to seven and nine, respectively, to have a more thorough estimation of the safety of the investigational regimen. None of the patients treated at dose levels I–III developed DLTs (Table 2

**Table 2:** Haematological and nonhaematological adverse events per dose level in cycles one and two and in subsequent cycles (worst per patient)

		**No. of patients/NCI-CTC grade**																									
**Dose level**	**Patients (*n*)**	**Anaemia**				**Leucocytopenia**				**Thrombocytopenia**				**Nausea/vomiting**			**Stomatitis/mucositis**			**Diarrhoea**			**Creatinine**		**HFS**		
		1	2	3	4	1	2	3	4	1	2	3	4	1/2	3	4	1/2	3	4	1/2	3	4	1	2	1	2	3
*Cycles 1/2*																											
I	3	–	–	–	–	–	–	–	–	–	–	–	–	3	–	–	–	–	–	1	–	–	–	–	1	–	–
II	7	3	1	–	–	1	–	–	–	3	–	–	–	2	–	–	–	–	–	1	–	–	2	–	2	1	–
III	9	–	2	–	–	–	–	–	–	2	–	–	–	4	–	–	3	–	–	3	–	–	–	–	–	1	–
IV	30	8	8	3	–	7	6	1	–	10	3	1	–	14	–	–	2	1	–	8	–	–	2	1	3	–	1
																											
*Cycles 3/4*																											
I	2	–	–	–	–	–	–	–	–	–	–	–	–	–	–	–	1	–	–	1	–	–	–	–	1	1	–
II	4	–	2	1	–	1	–	1	–	2	–	1	–	–	–	–	–	–	–	1	–	–	–	–	–	–	–
III	4	–	–	–	–	–	–	–	–	1	–	–	–	3	–	–	–	–	–	1	–	–	–	–	–	–	–
IV	15	4	3	–	–	2	1	1	–	3	3	2	–	1	1	–	1	–	–	1	–	–	–	1	1	2	–

Creat=creatinine; HFS=hand–foot syndrome.

). Furthermore, none of the first six patients treated at dose level IV had dose-limiting side effects, as well, and consequently a total of 34 patients were recruited at this dose level receiving a total of 124 treatment cycles. Of these patients, four were not evaluable for the previously mentioned reasons. Two of these patients formally had serious adverse events (port infection (*n*=1) and ileus due to tumour-related bowel obstruction (*n*=1)) clearly not related to the study treatment. Of the remaining 30 patients, two developed DLTs: neutropenic fever (*n*=1) and mucositis grade III (*n*=1). Thus, as foreseen by the protocol, a DLT rate of >17% could be excluded with a power of 80% and an *α*-value of 0.2.

**Figure 1:**
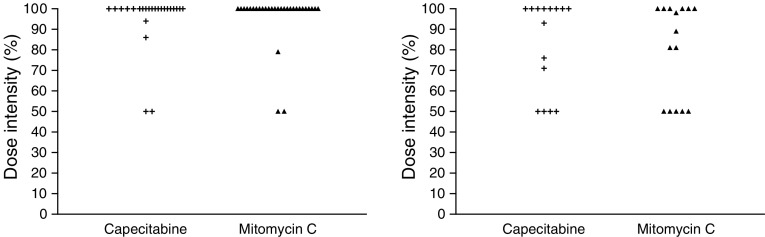
Dose intensity (%) in patients treated at the recommended dose during cycles one/two (*n*=30), and three/four (*n*=15).

Dose intensity was calculated for all patients treated at the recommended dose (level IV). Taking all 30 patients into account, a median dose intensity of 100% was reached during cycles one and two for each drug (Figure 1

). Postponement of treatment (which did not exceed 7 days in each case) was required during cycles one and two in only four patients. In addition, 15 patients (50%) received a third and fourth cycle of chemotherapy. The calculated median dose intensities for these patients in cycles three and four were capecitabine 100% and mitomycin C 89%. Seven of these 15 patients had a therapy delay during cycles three and four, with a median delay of 8 days (range 7–28 days). Three patients continued therapy and received at least five cycles of treatment.

At the recommended dose level, the mean cumulative dose of mitomycin C was 56 mg (median 47 mg; range 18–120 mg). At this level, 75% of the 30 patients received more than 36 mg of mitomycin C and 25% received more than 76 mg.

### Safety

Table 2 summarises haematological and nonhaematological adverse events during the first and second cycles (the time period for the determination of DLTs, i.e. days 1–42) and cycles three and four. No DLTs occurred at dose levels I–III. At dose level IV, a total of 30 patients were evaluable for safety. Leukopenia (*n*=14), thrombocytopenia (*n*=14), nausea/vomiting (*n*=14) and diarrhoea (*n*=8) were the most commonly observed adverse events during cycles one and two, while grade III events were observed in only four patients. Among 15 patients treated during cycles three and four, eight suffered from thrombocytopenia with a tendency towards higher grades of adverse events (grade 1, *n*=3; grade 2, *n*=3; grade 3, *n*=2) compared with cycles one and two. Both patients with grade 3 thrombocytopenia had been pretreated with cisplatinum for several months. One patient developed thrombocytopenia after a cumulative dose of 20 mg m^−2^ mitomycin C starting on day 30 and another patient after 30 mg m^−2^ (starting on day 55). However, no episodes of thrombocytopenic bleeding were reported. Grade 3 stomatitis/mucositis was observed in one patient and hand–foot syndrome (HFS) was seen in four patients at dose level IV during cycles one and two, although only one patient developed grade 3 HFS. No prophylactic measures against HFS (e.g. vitamin B6) were in place.

Slight elevations of serum creatinine were seen in five out of 49 (10%) patients evaluable for safety (grade 1, *n*=4; grade 2, *n*=1). No evidence of mitomycin C-induced haemolytic–uraemic syndrome was documented.

### Antitumour activity

Of the 27 patients with metastatic colorectal cancer enrolled, 25 were evaluable for antitumour response. These patients had received a median of three lines of prior chemotherapy, including 5-FU and either irinotecan or oxaliplatin or both. In two patients, tumour remissions were observed (8%; 95% confidence interval (CI) 0–19%). One patient each was refractory to two or three prior regimens, respectively. Seven patients (28%, five of whom were refractory) had SD following treatment. The median progression-free survival for all patients with colorectal cancer was 2.0 months, for patients with clinical benefit (PR and NC) 3.0 months (range 2.0–>7.6 months).

Of the 14 patients who had metastatic gastric cancer, 11 were evaluable for antitumour activity. Tumour shrinkage was noted in two of these patients (18%; 95% CI 0–45%), fulfilling the criteria for PR in one relapsed patient. Four patients (36%) had SD with a mean time of SD of 2.8 months (range 2.2–3.7 months). The median progression-free survival for all patients with gastric cancer was 2.3 months, for patients with clinical benefit 3.0 months (range 1.4–4.2 months). Furthermore, two patients with refractory pancreatic cancer had SD (lasting 3.4 and 3.2 months, respectively) and two patients with squamous cell carcinoma of the oesophagus had remissions lasting for 3.8 and >5.8 months.

## DISCUSSION

Our current findings indicate that mitomycin C at a dose of 10 mg m^−2^ on day 1 in combination with capecitabine 1000 mg m^−2^ twice daily on days 1–14 every 3 weeks is both effective and well tolerated, with an adverse event profile similar to capecitabine monotherapy (Cassidy *et al*, 2002). Dose escalation in this phase I study continued to the highest dose level (IV) without the occurrence of DLTs at lower dose levels. At dose level IV, only two patients experienced a DLT. Gastrointestinal adverse events were infrequent, although haematological adverse events, predominantly thrombocytopenia, were more pronounced.

A total of 15 patients received at least four cycles of chemotherapy. During cycles three and four, higher grades of thrombocytopenia were noted. This shift in toxicity was expected because delayed thrombocytopenia is known to develop after prolonged therapy with mitomycin C. Mainly because of this adverse event, an observation period of 6 weeks (two courses of chemotherapy) for the development of DLTs was required by the protocol. Nevertheless, dose intensity for mitomycin C was maintained at 100% during the first two cycles (*n*=30 patients), and 89% during cycles three and four (*n*=15 patients). In addition, there were no episodes of haemolytic uraemic syndrome or pulmonary toxicities during the study.

Our safety data are comparable to findings from two studies published as abstracts by Rao *et al* (2003) and Watkins *et al* (2003) from the Royal Marsden Hospital, UK. The authors reported on patients with metastatic colorectal cancer (first line, *n*=55; third line, *n*=31), who received capecitabine 1250 mg m^−2^ twice daily for 14 days, every 3 weeks, in combination with mitomycin C as a bolus of 7 mg m^−2^ on day 1, every 6 weeks. The proportion of patients experiencing grade 3/4 adverse events with this low-dose mitomycin C/high-dose capecitabine regimen were as follows (first line *vs* third line): thrombocytopenia (3.6% *vs* not reported); neutropenia (3.7 *vs* 1.8%); anaemia (0 *vs* 7.4%); diarrhoea (14.3 *vs* 3.9%); nausea/vomiting (1.8 *vs* 11.5%). In addition, the safety profile of the same schedule evaluated in 27 patients with colorectal cancer treated on a compassionate use basis after failure of 5-FU, irinotecan and oxaliplatin (Harba *et al*, 2003) was reported to be in the same range as in the Royal Marsden studies and in our phase I study.

The patients in our study were heavily pretreated with a median of three regimens of prior chemotherapy for colorectal cancer and one regimen for gastric cancer. Nevertheless, we observed tumour regressions in patients with either tumour type. The response rate of 8% and SD rate of 28% observed in the 25 patients with colorectal cancer is almost identical to that reported by Harba *et al* (response rate 10%, SD 30%), but somewhat lower than that from the Royal Marsden study (response rate 22%, SD 57%) (Rao *et al*, 2003), both of which studied the combination in the salvage setting. Nevertheless, the patients included in the latter study had not received prior oxaliplatin. Of note are the results from the Royal Marsden study examining capecitabine/mitomycin C as first-line chemotherapy, including an overall response rate of 40% (95% CI, 26.4–54.0%), a median survival of nearly 15 months and a median TTP of 7.2 months (Watkins *et al*, 2003). These findings with capecitabine/mitomycin C as front-line therapy in metastatic colorectal cancer compare well with 5-FU/irinotecan or 5-FU/oxaliplatin combination studies (de Gramont *et al*, 2000; Douillard *et al*, 2000; Saltz *et al*, 2000). Examining the results from all four trials on capecitabine/mitomycin C therapy reveals that a response rate of approximately 40% can be achieved in chemonaïve patients, while every round of pretreatment appears to reduce the likelihood of response by an estimated 10% (response rate of 10% after 5-FU/irinotecan/oxaliplatin (Harba *et al*, 2003. present study), response rate of 20% after 5-FU/irinotecan (Rao *et al* (2003)), response rate of 30% after 5-FU monotherapy).

Meanwhile, data on patients with advanced biliary tract cancer have been published that indicate that the mitomycin C and capecitabine combination regimen (mitomycin C 8 mg m^−2^ on day 1 plus capecitabin 1000 mg m^−2^ twice daily on days 1–14, every 4 weeks) may also be a promising therapeutic option for this type of cancer (Kornek *et al*, 2004).

In conclusion, we have established a dosing schedule for the combination of capecitabine and mitomycin C that is both well tolerated and effective for the treatment of patients with previously treated advanced/metastatic colorectal and gastric cancer. Activity was also seen in pancreatic cancer and squamous cell carcinoma of the oesophagus. This regimen may be considered for patients with colorectal cancer after pretreatment with 5-FU, irinotecan and oxaliplatin, but it is also likely to be a valuable, cost-saving and convenient treatment option even in earlier stages of colorectal cancer chemotherapy. Our results in gastric cancer patients, together with our earlier reported experience with infusional 5-FU/mitomycin C combination therapy in this type of cancer (Hartmann *et al*, 2003; Hofheinz *et al*, 2002), suggest that the capecitabine/mitomycin C regimen could be considered as an alternative regimen for patients not suitable for cisplatin-based therapy.
